# Supplementary material for: High Resolution Analysis of Proteome Dynamics during Bacillus subtilis Sporulation
Source: Int J Mol Sci. 2021 Aug 28;22(17):9345. doi: 10.3390/ijms22179345 (PMC8431406; doi:10.3390/ijms22179345)
Supplement: Supplementary file 1 [file ijms-22-09345-s001.zip › Supplementary figures.pdf]

High resolution time-lapse proteomic analysis of *Bacillus subtilis* sporulation

Zhiwei Tu[1,2], Henk L. Dekker[2], Winfried Rosenboom[2], Bhagyashree N. Swarge[1,2], Peter Setlow[3], Stanley Brul[1], Gertjan Kramer[2,*]

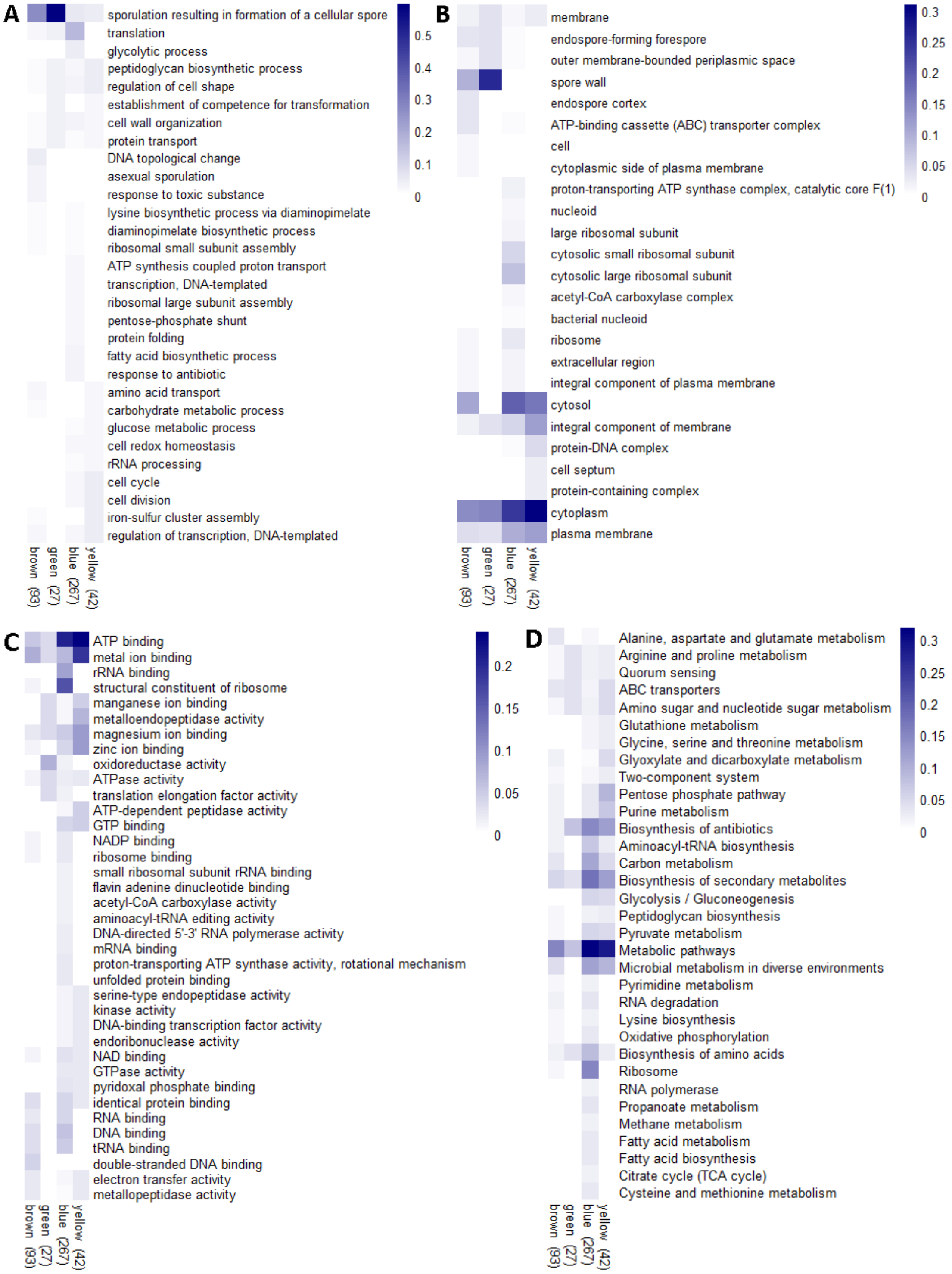

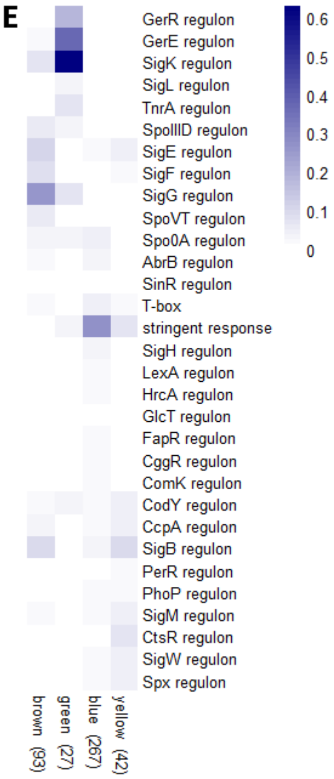

Figure S1. Heatmap visualization of GO (gene ontology) annotation (A, B and C), KEGG pathway (D) and transcriptional regulons (E). (A) GO biological processes. (B) GO cellular compartments. (C) GO molecular functions. The progressively darker blue indicates the fractions of the module proteins belonging to the categories labeled on the right of the heatmap. GO categories, KEGG pathways and Regulons with more than 3 proteins in total are shown in the heatmap. Module size is displayed in the brackets following the module name.
